# PROTOCOL: Technology‐based and digital interventions for intimate partner violence: A meta‐analysis and systematic review

**DOI:** 10.1002/cl2.1132

**Published:** 2021-01-14

**Authors:** Chuka Emezue, Tina L. Bloom

**Affiliations:** ^1^ Sinclair School of Nursing University of Missouri‐Columbia Columbia MO USA; ^2^ Sinclair School of Nursing University of Missouri‐Columbia Columbia MO USA

## Abstract

**Introduction:**

Studies show digital interventions such as mobile and wireless platforms (e.g., smartphone apps, text messaging) and web‐based platforms (online support groups and telehealth services) can improve the mental health outcomes for victims/survivors of partner abuse. Depression, posttraumatic stress disorder (PTSD), and anxiety are three to five times higher among victims than nonvictims and are thus popular targets of digital interventions. Even then, the evidence is scant. The current review uses both narrative and quantitative (meta‐analysis) techniques to present extensive evidence on the effects of intimate partner violence (IPV) digital interventions on the mental health outcomes among survivors of partner violence across all genders and ages, specifically, depression, anxiety, and PTSD. This is the first meta‐analysis on IPV‐related mental health outcomes targeted by digital interventions.

**Objectives:**

To synthesize current evidence on the intervention and treatment effects of digital and technology‐based interventions (mHealth and eHealth) addressing IPV mental health outcomes (depression, anxiety, and PTSD) among survivors of IPV. This study's research questions are as follows: (a) What are the overall average treatment effects of IPV digital interventions on IPV survivors' mental health outcomes? (b) Do these mental health outcomes vary based on methodological study designs, sample characteristics, and intervention characteristics?

**Methods:**

An extensive search strategy will be utilized to find qualifying studies. Various electronic bibliographic databases will be searched for studies since 2009 (coinciding with the onset of mobile health interventions). Other databases, such as government databases, grey literature databases, trial registers, specialty journals, and citations in other studies will be searched. Also, we will search “grey databases,” such as Google Scholar. Ethical and safety concerns preclude the randomization of IPV survivors to specific intervention conditions. Therefore, we will not exclude studies based on a lack of random assignment. Studies will be full‐text accessible, published in any language (translatable into English). We will also contact researchers where needed data is missing in their report. Neither language, study location, nor study settings will be a limiter for searches. Keyword and MeSH headings will be used. Effect sizes (Hedges' g) will be estimated with a Type I error rate set at an alpha of .05.

**Results:**

All studies will measure IPV‐related mental health as an outcome and provide outcome data to calculate effect sizes for PTSD, anxiety, depression, and victimization (physical, psychological, and sexual violence).

**Conclusion:**

Digital interventions may clinically reduce depression, anxiety, PTSD, and IPV victimization. Summary effect sizes ranging from small to large will signal the usefulness of digital interventions to IPV survivors contending with common mental health issues. Future studies beyond this one may identify other active intervention ingredients of digital interventions, best modes of delivery, and guidelines to increase their feasibility and acceptability.

## 1 BACKGROUND

### 1.1 The problem, condition, or issue

Intimate partner violence (IPV) remains a public health, social justice, and human rights issue. Being a form of domestic violence, IPV involves physical violence, sexual violence, stalking, or psychological harm by a current or former partner or spouse regardless of sexual intimacy, relationship type, gender, or sexual identity (McFarlane et al., 2002; WHO, 2019). Whereas domestic violence broadly encompasses several forms of violence such as child or elder abuse, or abuse by any member of a household, our focus in this meta‐analysis is on violence between partners or spouses, to include premeditated and systematic patterns of coercive, assaultive, threatening behaviors including but not limited to physical abuse, sexual violence, coercive control, verbal/psychological aggression, intimidation, digital abuse, isolation, stalking, financial abuse, reproductive coercion, and femicide—among other forms of abuse (Ali, Dhingra, & McGarry, 2016; Black et al., 2011).

On average, about 24 people a minute are physically assaulted by an intimate partner in the United States, approximating more than 10 million US women and men annually (Breiding, Chen, & Black, 2014). Over 25% of US adult women and one in three women aged 15–49 years worldwide are adversely impacted by physical, psychological, sexual violence, and stalking in their lifetime (Breiding et al., 2014), with prevalence ranging from 24% in Serbia and Montenegro to 71% in rural Ethiopia (García‐Moreno et al., 2013; Keynejad, Charlotte, & Howard, 2020). A long list of adverse sequelae for women's physical, mental, sexual, reproductive, and psychosocial health is associated with IPV experiences (see notes in Section 1.3; Black et al., 2011, Breiding et al., 2014; McFarlane et al., 2002). Several meta‐analyses suggest links between IPV experiences and adverse mental health disorders (Lagdon, Armour, & Stringer, 2014). For example, IPV survivors report depression and anxiety three to five times higher than nonsurvivors (Beydoun, Beydoun May, Kaufman, Lo, & Zonderman, 2012; Lagdon et al., 2014). Between 30% and 80% of survivors meet the criteria for posttraumatic stress disorder (PTSD), with symptoms including (but not limited to) persistent intrusive memories, persistent avoidance/attachment, emotional numbing, flashbacks/nightmares, victim hypervigilance, and persistent symptoms of physiological arousal (American Psychiatric Association, 2013; Nathanson, Shorey, Tirone, & Rhatigan, 2012).

The physiological impact of IPV span from cardiovascular (e.g., hypertension and chest pain), gastrointestinal (chronic irritable bowel syndrome, appetite loss, eating disorders), reproductive (injuries to genitalia), musculoskeletal issues (e.g., craniofacial injury), and can affect the nervous system typically through traumatic brain injury (TBI; Akinsulure‐Smith, Chu, Keatley, & Rasmussen, 2013; García‐Moreno et al., 2013; Gonçalves & Matos, 2016; Liles et al., 2012; Peterson et al., 2018; Raj & Silverman, 2002). With an alarming annual price tag of $3.6 trillion (US 2014 rates) in terms of Disability‐Adjusted Life Years, the lifetime per survivor expense for women is $103,767 and men ($23,414)(Peterson et al., 2018). IPV's socioeconomic costs involve a matrix of legal, criminal, personal, and medical expenses (59% of total cost) putting lifetime per victim cost at an average of $81,960 (Peterson et al., 2018, p. 438).

Emerging literature suggests the promise of digital and technology‐based interventions (or *digital interventions* for short) to complement traditional or human‐delivered modalities for supporting survivors of IPV. Compared with traditional interventions, digital interventions are designed to meet survivors where they are by bridging provider‐client coverage gaps linked to health system challenges and inequalities. At the user‐level, digital interventions prioritize privacy, confidentiality, and user safety while offering reliable and personalized real‐time care to meaningfully and clinically improve health and wellbeing (Glass, Eden, Tina, & Nancy, 2010; Glass et al., 2017).

IPV digital interventions utilize a confluence of technologies, such as mobile and wireless platforms (e.g., smartphone apps, text messaging) as well as web‐based platforms (online support groups and telehealth services) to improve survivor outcomes (Bloom, Gielen, & Glass, 2016; Debnam & Kumodzi, 2019; Koziol‐McLain et al., 2018; Klevens, Sadowski, Kee, Trick, & Garcia, 2012; May, Mort, Williams, Mair, & Gask, 2003; Young, Pruett, & Colvin, 2018). Digital interventions can also support routine screenings of reproductive‐age women (14–46 years) as recommended by the US Preventive Services Task Force (USPSTF; Moyer, 2013). Digital interventions are of benefit to the hard‐to‐reach low‐income survivors of partner abuse, especially in health provider scarcity areas where IPV victimization may overlap with established social determinants of violence (e.g., socioeconomic status, immigration status, gender norms, disabilities, age, and race/ethnicity) (Akinsulure‐Smith et al., 2013; Bui & Morash, 2008; García‐Moreno et al., 2013; Gonçalves & Matos, 2016; Liles et al., 2012; Peterson et al., 2018; Raj & Silverman, 2002). Digital and technology‐based interventions to improve mental health outcomes among victims‐survivors of IPV are the focus of this systematic review and meta‐analysis.

#### 1.1.1 What are digital and technology‐based interventions?

Digital and technology‐based interventions include eHealth (or electronic health), mobile (or mHealth) platforms, and telehealth platforms. According to the World Health Organization, eHealth platforms include “information and communications technology in support of health and health‐related fields.” (WHO, 2019). Similarly, mhealth platforms (a subset of eHealth) incorporate “mobile wireless technologies for health” and constitute a branch of eHealth involving mobile devices like smartphones and tablets to deliver or use health care services, exchange clinical information, and collect data. Telehealth platforms, on the other hand, encompass the use of ICT (or information and communication technologies) by healthcare practitioners to provide healthcare services, facilitate the secure sharing of health information, allowing patients to communicate health‐related concerns such as prevention, diagnosis, treatment, and remote follow‐up while addressing logistics and distance concerns (WHO, 2019). For this review and meta‐analysis, we define *digital interventions* broadly to include any use of technology to deliver information, treatment, therapy, or psychosocial support to improve the mental health of survivors of partner violence.

The proliferation of digital interventions align with rapid developments in patient technology literacy, device ownership, and device features (i.e., portability, simplicity, affordability, and esthetics). Their simplicity and convenience have prompted a growing reliance on them for the delivery of health and behavioral interventions used in ecologically valid ways, especially for round‐the‐clock, safe, and confidential patient‐to‐provider contact (Anderson‐Lewis, Darville, Mercado Rebeccah, Howell, & Di Maggio, 2018; Ranney, Choo, Spirito, & Mello, 2013). Importantly, technology‐based interventions (whether in the form of a smartphone app, social media forum, web‐based, or telehealth intervention) have found widespread use given the impact of COVID‐19 on the usual support systems for survivors of partner violence (Emezue, 2020). Several existing and in‐progress digital interventions are now being developed and adapted to meet specific outcomes among survivors (Koziol‐McLain et al., 2018).

Evidence of the intervention effectiveness and treatment effects of IPV digital interventions addressing survivors' mental wellbeing is still emerging. This lack of evidence is partially due to the novelty of digital IPV interventions and our limited knowledge of the impact of digital IPV interventions on mental and psychosocial outcomes. The importance of IPV digital interventions, the need to improve mental health, the proliferation of randomized trials pilot‐testing IPV digital interventions, and the diversity of intervention evidence provide a strong rationale for summarizing current findings.

The issues above bear significant implications for intervention engagement, uptake, and overall treatment effects. Findings from this meta‐analysis will be crucial to the work now being done by interdisciplinary IPV digital health teams comprising violence preventionists, researchers, advocates, survivors, and platform developers. Moreover, a comparative assessment of clinically meaningful treatment effects on specified outcomes (i.e., mental health) will convince funders and policymakers to support adaptation, validation, and scale‐up efforts. This will encourage the integration of mHealth/eHealth interventions into relevant higher‐level universal screening systems to align with calls for routine screenings of reproductive‐age women (14–46 years) as recommended by the USPSTF (Moyer, 2013).

For researchers, findings bear implications for the evidence‐based development, pilot‐testing, adaptation, and deployment of digital interventions that support the mental health and wellbeing of IPV survivors. This protocol presents a plan for a meta‐analysis and narrative review that will investigate the effects of digital interventions on IPV mental health outcomes to see if intervention effects are different in clinical and meaningful ways.

#### 1.1.2 Digital interventions addressing IPV mental health outcomes

Digital technologies are often used with traditional modalities like group counseling, face‐to‐face therapy, psychosocial‐behavioral therapies, psychoeducation, mindfulness, and safety planning training, making them an adjunct layer of support for IPV survivors. In light of these applied uses, calls for rigorously‐tested evidence‐based IPV digital interventions have matched the growing efficacy of digital interventions in other health behavior domains. Several meta‐analyses focusing on alcohol abuse, physical activity, depression, anxiety, bullying, diabetes self‐management, cannabis, and tobacco use, teen pregnancy, and smoking cessation across diverse cohorts show the promise of digital interventions (Anderson‐Lewis et al., 2018; Firth et al., 2017; Free et al., 2010; Li, Theng, & Foo, 2014; Liang et al., 2011; Khadjesari, Elizabeth, Catherine, Suzanne, & Christine, 2011; Luk et al., 2019; Montgomery, Robinson, Seaman, & Haeny, 2017; Ybarra, Prescott, & Espelage, 2016).

Consistent with this viewpoint, a series of meta‐analyses show increased odds of experiencing adverse mental health with IPV victim‐survivors (Nathanson et al., 2012). Depression, PTSD, and anxiety are three to five times higher among IPV survivors than nonvictims of IPV (Golding, 1999; Lagdon et al., 2014). Another meta‐analysis corroborates these findings, indicating women reporting IPV experiences had two to three times the risk for major depressive disorder (MDD) and twice the risk of elevated depressive symptoms and postpartum depression compared to non‐IPV women (with no partner violence experience) (Beydoun et al., 2012). Reports of moderate to strong positive correlations between IPV and depression are replete in the IPV literature (Beydoun et al., 2012; Mechanic, Weaver, & Resick, 2008; Tol et al., 2019).

Since survivors of IPV exhibit PTSD symptomologies (51%–75% of female victim‐survivors) co‐occurring with depression and anxiety, mental health outcomes remain veritable targets for IPV prevention (Ellsberg et al., 2015; Gevers & Dartnall, 2014; Lagdon et al., 2014; Sharhabani‐Arzy, Amir, & Swisa, 2005). Treating IPV‐related mental health disorders is also an effective strategy in preventing the onset (primary prevention) and future reoccurrence (tertiary prevention) of IPV (Tol et al., 2019). Left untreated, mental health disorders interfere with treatment uptake, create barriers to holistic treatment, impact the general quality of life (Johnson & Zlotnick, 2009; Johnson, Zlotnick, & Perez, 2011; Nathanson et al., 2012), and can create an environment of chronic polyvictimization and multimorbidity (Nathanson et al., 2012).

Having shown promise, partner violence survivors are becoming receptive to trauma‐informed technology‐based interventions (Glass et al., 2017; Koziol‐McLain et al., 2018; Littleton, Grills, Kline, Schoemann, & Dodd, 2016). Specifically, where it is perceived that digital interventions can moderate barriers to access, provide safe options to leave or navigate an abusive relationship, be tailored to unique social ecologies, and mitigate IPV‐related burden (Glass et al., 2017). Digital interventions mediate some of the shortcomings of using traditional interventions. For example, the cost of care, nonconfidentiality, poorly trained health providers, geographical inaccessibility, stigma with requesting care (e.g., rape services), and socio‐geographical diversity are considered in the design and planning of web‐based and mobile‐based IPV interventions directly to victims in need of social, informational, and material support (Constantino et al., 2015; Eden et al., 2015; Ford‐Gilboe et al., 2017; Glass et al., 2010, 2017; Koziol‐McLain et al., 2018; Littleton et al., 2016).

### 1.2 The intervention

More than half a billion people worldwide have downloaded a health‐related smartphone application (app; Dorsey, McConnell, Shaw, Trister, & Friend, 2017), enabling patients to produce, monitor, and regulate their health data (Anderson‐Lewis et al., 2018; Dorsey et al., 2017; Klasnja & Pratt, 2012). Digital interventions are often designed to target mental health outcomes such as depression (Braithwaite & Fincham, 2011; Glass et al., 2017), suicidal ideation (considering or planning suicide), substance use disorders, anxiety disorders, and posttraumatic stress disorders, and PTSD (Braithwaite & Fincham, 2011; Constantino et al., 2015). Actual reductions in victimization (i.e., positive treatment effect) can also be a primary target of digital interventions.

Beyond mental health, IPV digital interventions may promote psychosocial wellbeing by improving emotional and instrumental social support, safety planning, and health‐related quality of life (HRQoL; Constantino et al., 2015). Even so, the survivors' decisional conflict (or uncertainty about the best course of action), general distress (e.g., feelings of hopelessness, anger, and disempowerment), and ongoing IPV exposure have become targets of IPV digital interventions (Glass et al., 2017; Koziol‐McLain et al., 2018; Littleton et al., 2016). In this way, digital interventions work by offering ongoing social and emotional support, increasing safety planning, improving risk awareness of abuse, severity, providing psychoeducation (such as identifying red flags of abuse), and triaging survivors to trusted care based on the survivor's unique social and personal contexts.

Other targetable outcomes are psychosocial and nonclinical and may include survivor‐defined empowerment, self‐efficacy to create and execute a safety plan, minimize decisional conflict (or uncertainty about how to navigate an abusive relationship), and improve safety planning for significant others (e.g., children, pets). Current IPV digital interventions combine traditional modalities to reach diverse user groups with comprehensive and personalized care. Researchers, advocates, and interventionists administer digital interventions in various settings (clinical, school, shelter, community, and online communities).

#### 1.2.1 An example of an IPV digital intervention

The myPlan app (www.myplanapp.org) serves primarily as a decision aid to help survivors make informed decisions about their safety and wellbeing. Its theoretical underpinning stems from the decisional conflict model using an empowerment approach to raise awareness of violence severity and then create a safety plan (Dutton, Goodman, & Bennett, 1999; Eden et al., 2015; Glass et al., 2008; Glass et al., 2017). In sum, the myPlan app (a) educates the survivor on relationship red flags; (b) estimates their level of danger (i.e., abuse severity) and risk for fatality using a weighted scoring protocol called the Danger Assessment (DA) tool (Campbell, 2002; Campbell & Glass, 2009); (c) estimates priorities for safety (e.g., child's welfare) using pairwise comparisons of priorities; (d) creates a checklist of tailored safety planning; (e) designs a tailored safety plan based on the survivor's level of danger and priorities; and ultimately (f) triages the victim‐survivor to trusted resources based on some consideration of their social ecology (Glass et al., 2010).

The myPlan app was developed iteratively in two phases—via (a) formative development with experts, advocates, and survivors; and (b) pilot testing with survivors (all female) in domestic violence shelters and support groups (Glass et al., 2010). The myPlan intervention was first pilot‐tested as a computerized version, hypothesizing improvements in the survivor's decisional conflict (Glass et al., 2010). Following a 12‐month follow‐up, the authors suggest this safety decisional aid reduced decisional conflict (*p* = .014, 95% confidence interval [CI]) and increased the survivor's likelihood of creating a safety plan for themselves and their children with the option of leaving an abusive relationship if desired. Following the development of the myPlan app, several adaptions of the app have been tested with at‐risk sexual and ethnic minority groups, including Maori‐women (Koziol‐McLain et al., 2018), immigrant women (Sabri et al., 2019), LGBTQIA+ people, college women (Bloom et al., 2016), college women and their friends (Alhusen, Bloom, Clough, & Glass, 2015), multilanguage versions available in Spanish and English (Eden et al., 2015; Glass et al., 2017) with several ongoing country‐specific adaptations and clinical trials.

### 1.3 Description of the condition

As the most common form of domestic violence, IPV remains a public health, social justice, and human rights issue. IPV prevalence data are commonly described in terms of the frequency of occurrence (episodic or chronic abuse), type, severity, and lethality of abuse perpetrated by an intimate partner—current or past (Black et al., 2011; García‐Moreno et al., 2013). Of note, IPV predominantly impacts female and femme adolescents and adults. While the majority of perpetrators are men (or male‐identified), IPV occurs irrespective of gender or sexuality, across the lifespan (impacting adolescents and adults), and across ethno‐racial groups (Akinsulure‐Smith et al., 2013; García‐Moreno et al., 2013; Gonçalves & Matos, 2016; Liles et al., 2012; Malley‐Morrison & Hines, 2004; Peterson et al., 2018; Raj & Silverman, 2002). Victimization is notably higher among women and girls from minoritized groups, such as are immigrants, refugees, and asylum seekers, as well as victims‐survivors with disabilities, living in rural areas, elderly women, women and girls in low‐ or middle‐income regions, First Nation and Indigenous Women, LGBTQIA+ people, and teenagers in dating relationships (Black et al., 2011; Bui & Morash, 2008; García‐Moreno et al., 2013; Koziol‐McLain et al., 2018).

Lifetime and past‐year prevalence estimates are commonly reported estimates of partner violence. Over 25% of adult US women and 30% of women worldwide (or about 938 million women) are affected by physical and sexual forms of IPV across a lifetime (Breiding et al., 2014). The biopsychosocial wellbeing of victim‐survivors is often under threat, with worse case outcomes resulting in femicide (or the intentional murder of female partners). Intimate male partners commit over 38% of all female homicides (Sabri, Campbell, & Dabby, 2016). In the US, 25% of women and 14% of men report severe forms of IPV, even with underreporting and under‐representation of some vulnerable groups (Black et al., 2011). Of note, adult IPV differs from IPV in adolescence, referred to as Teen Dating Violence (TDV). Approximately one in four (or 25%) of US high schoolers report sexual or physical victimization experiences in the current or past year, and another two‐thirds report psychological victimization (De La, Polanin, Espelage, & Pigott, 2017; Decker, Silverman, & Raj, 2005; Foshee et al., 2012, 2014; Vagi et al., 2013). The highest exposure to TDV occurs between age 16–18 (Breiding et al., 2014; Coker et al., 2015; Foshee et al., 2014; Haynie et al., 2013), leading to poor academic performance, gang involvement, unintended pregnancies, adolescent homicide (5,319 US youth in 2016, see Adhia, Kernic, Hemenway, Vavilala Monica, & Rivara Frederick, 2019). Other adverse outcomes for adolescents include depression, self‐harm, suicidality, health risk behaviors (e.g., HIV risk behaviors), and substance dependence for coping (Adhia et al., 2019; Coker et al., 2015; Haynie et al., 2013; Miller‐Perrin, Perrin, & Renzetti, 2017; Nathanson et al., 2012). Moreover, TDV remains strongly associated with future violence in adulthood when it is harder to manage (Miller‐Perrin et al., 2017; Pensak, 2015).

Adverse sequelae of IPV span biopsychosocial areas of impact to include a host of cardiovascular, gastrointestinal (e.g., vaginal bleeding and urinary tract infections), reproductive/gynecological issues (e.g., injuries to genitalia, sexually transmitted infections), as well as musculoskeletal issues (e.g., craniofacial injuries; Afifi, Makhoul, El Hajj, & Nakkash, 2011; Campbell, 2002). Other problems include central nervous system conditions (e.g., traumatic brain injuries, headaches, fainting, or seizures), and the development of chronic diseases, pain, and disabilities (Afifi et al., 2011; Campbell, 2002; Coker et al., 2015). Further, IPV mental outcomes include generalized anxiety disorders (such as PTSD), suicidality, phobias, self‐harm, social anxieties, personality, sleep and eating disorders (e.g., appetite loss; Black et al., 2011, Breiding et al., 2014; Campbell, 2002; Glass et al., 2008; Glass et al., 2017; Tjaden & Thoennes, 1998, 2000). The social epidemiology of IPV is not just a problem for intimate partners. Its adversity significantly impacts the child(ren) who witness IPV, the family as a unit, and the community.

### 1.4 Description of the intervention

This meta‐analysis focuses on digital and technology‐based interventions addressing IPV‐related mental health outcomes, whether as a primary or secondary intervention priority for IPV survivors. We use a broad definition of digital intervention as any use of technology to deliver material treatment or psychosocial support to improve the mental health of survivors of partner violence, as a primary or secondary aim. These digital intervention are deployed via mHealth, eHealth, telehealth, and web‐based modalities to augment traditional interventions and are typically provided to survivors in clinical and community‐based settings (Trabold, McMahon, Alsobrooks, Whitney, & Mittal, 2020; Warshaw, Sullivan, & Rivera, 2013). IPV digital interventions, however, find applied use for IPV diagnosis, consultation, psychoeducation, safety planning, referral‐to‐care, and ongoing emotional and behavioral support across gender and age groups. Studies with digital interventions meeting the above description, with or without any comparator/control groups are relevant to this meta‐analysis. Where there are control groups, controls may be randomized to alternative forms of IPV interventions, including nondigital methods (e.g. paper and pencil surveys, checklists), traditional modalities (e.g. counseling, advocacy group sessions, standard shelter services, home visitation), and other intervention‐control mechanisms (e.g., usual care [UC], or waitlist).

### 1.5 How the intervention might work

Digital interventions may work in the following ways.

1. Offering ongoing or one‐time social and emotional support.
2. Increasing individualized safety planning and risk assessment of frequency, severity, and types of violence.
3. Providing information and psychoeducation to improve informed decision‐making.
4. Improving evidence gathering and documentation.
5. Reducing exposure to IPV (triage to local and trusted services).
6. Improving mental health outcomes (depressive disorders, generalized anxiety disorder, and PTSD).
7. Address co‐occurring morbidities linked to IPV (e.g., housing and employment needs).
8. Triage survivors to trusted care based on the survivor's unique social and personal contexts.

Though not clearly explained in most studies, the mechanism of action for improvement on survivor mental health derives from the material and emotional support provided using evidence‐backed digital interventions to alleviate the effects of IPV, support safety planning, extend the reach of mental healthcare, and in turn contribute to short and long‐term reductions in mental health issues.

With the proliferation of mobile devices and the ubiquity of Internet coverage (even in some low‐income settings), IPV digital interventions are considered a cost‐saving, provider‐mediating, and scalable bargain. Particularly, where they augment conventional modalities by targeting a spectrum of health and psychosocial outcomes related to IPV victimization, including suicidality, depression, anxiety disorders, PTSD, social withdrawal, low self‐esteem and self‐efficacy, economic self‐sufficiency, and other sequelae (Campbell, 2002).

Of note, with IPV intervention design, the articulation of methodological and theoretical concepts differs widely across studies. Essential components of IPV digital interventions are driven by many concepts of systems design and victim empowerement paradigms (Trabold et al., 2020). Theoretical approaches, for example, use a strength‐based and empowerment‐focused approach to improve the autonomy, protection, and empowerment of survivors (e.g., Dutton's empowerment model) while taking into account the disparity in power, gender, and socio‐behavioral nature of partner violence (Dutton et al., 1999; Warshaw et al., 2013). Digital health interventionists from IPV borrow social and behavioral science from feminist theories (e.g., gender and power theory), social cognitive theory, family systems theories, theories of technology adoption, and theories of harm reduction, all embedded in a social‐ecological context (Lawson, 2012). Importantly, the involvement of survivors at all phases of the design of digital intervention has become essential. Several acceptability studies are published detailing user‐centered methodologies to ensure that survivors get exactly the type of digitalized services they require.

In terms of functionality, digital interventions strife to minimize user burden (e.g., low data burden, safe Internet access, interactive features, round‐the‐clock services) even as considerations for user safety and confidentiality remain foremost. Some are designed as chat spaces, virtual communities, or linked to online interactive websites (e.g., telecounseling). Some digital therapeutics are delivered using low‐data messaging platforms—a more pragmatic way to support low‐income survivors in no‐tech and low‐tech areas. Evidence‐based apps like the myPlan app use a combination of pairwise comparisons and checklist functions to determine risk level and create tailored safety plans. Other apps serve functional purposes, for example, victim notification apps like VINEmobile or regular messaging apps for client check‐ins.

Moreover, most traditional interventions happen after violence has occurred and so use downstream secondary and tertiary prevention approaches to reduce or stop IPV and provide necessary services to support survivors and their families (García‐Moreno et al., 2013; Hawk et al., 2017; Wood, Glass, & Decker, 2021). Digital interventions attempt to focus on different levels of prevention, and so this meta‐analysis focuses on primary, secondary, and tertiary prevention contexts.

### 1.6 Why it is important to do this review

The current proliferation of IPV digital intervention presents a strong rationale for synthesizing narrative and quantitative evidence on omnibus intervention and treatment effects of these types of interventions. COVID‐19 stay‐at‐home and social distancing mandates have made the need for digital therapies even more pertinent, fostering an increasing reliance on remote digital interventions to support IPV survivors and their children (Emezue, 2020). Even more important, the impact of moderators (changing risk levels due to the cyclical nature of IPV, survivor's age, pre‐existing technology literacy, socioeconomic status, polyvictimization, or multiple forms of chronic abuse) on the uptake and treatment effects of digital interventions are not fully understood by IPV researchers, giving urgency to this review and meta‐analysis.

To their merit, evidence shows that conventional (or nondigital) interventions decrease clinical anxiety, depressive symptoms, and PTSD by way of moderate‐to‐large effect sizes, offering both meaningful and clinically significant benefits to survivors (Warshaw et al., 2013). A recent review and meta‐analysis of traditional interventions compared the benefits of short‐term intervention to not receiving treatment (large omnibus effect sizes of Hedge's *g* = 1.02, see Arroyo, Lundahl, Butters, Vanderloo, & Wood, 2017), showing decreased PTSD symptoms, increased self‐esteem, reduced symptoms of depression and general distress, and increases in life functioning. Several systematic reviews also indicate that traditional psychosocial therapies involving cognitive‐behavioral therapy modalities also show continued promise for treating mental health disorders (Arroyo et al., 2017; Mendes, Mello Marcelo, Ventura, de Medeiros Passarela, & de Jesus Mari, 2008; Roberts, Kitchiner, Kenardy, & Bisson, 2009).

However, in the IPV digital intervention literature, this degree of empirical and clinical certainty (i.e., power and precision) is missing. A meta‐analyzed synthesis of their effect on mental health outcomes is imperative for reliable and scalable intervention planning, especially where victimization intersects with race/ethnicity, socioeconomic deprivation, and minority statuses, prompting reliance on alternative treatment/intervention methods (Trabold et al., 2020; Warshaw et al., 2013). The psychosocial and clinical benefits of targeting mental health outcomes for IPV survivors are widely published (Golding, 1999; Lagdon et al., 2014). Improved mental health outcomes have been linked to improved psychosocial functioning, emotional/material empowerment, safety behaviors, social participation, and higher HRQoL among IPV survivors. We also know from the systematic review and meta‐analysis by Keynejad et al. (2020) that psychological treatments for common mental illnesses among women in low‐income and middle‐income countries successfully treat anxiety in women when given by qualified clinicians, even if not targeted to that survivors, or targeting intimate partner abuse directly.

This is the first meta‐analysis on IPV‐related mental health outcomes targeted by digital interventions. While it is known at the study‐level that digital interventions improve survivor decisional certainty, safety behaviors (e.g., safety planning), and psychological outcomes for survivors of IPV, the extent to which they contribute to improvements in mental health outcomes remains unknown (Ford‐Gilboe et al., 2017). No conclusions about the effects of digital interventions on IPV mental health outcomes have yet to be made beyond a recently published systematic review (Anderson et al., 2019). There have been no attempts to cumulate the intervention (or treatment) effects of digital interventions on survivors' mental health, even though exploratory analyses of the IPV literature indicate that mental health outcomes such as depression, anxiety disorders, and PTSD are primary targets of intervention and are commonly indicated among adolescent and adult survivors in trial studies for IPV (Keynejad et al., 2020; Lagdon et al., 2014; Nathanson et al., 2012).

The current review uses both narrative and quantitative (meta‐analysis) techniques to present extensive evidence on the effects of IPV digital interventions on the mental health outcomes among survivors of partner violence across all genders and ages, specifically, depression, anxiety, and PTSD, as operationally defined by the Diagnostic and Statistical Manual of Mental Disorders (according to American Psychiatric Association, 2013). Findings from this meta‐analysis are significant in three ways.

1. For service providers seeking to identify the immediate and distal benefits/risks of digital approaches designed to support the emotional wellbeing of adult and adolescent survivors seeking evidence‐based treatment models, the findings will include evidence‐to‐decision frameworks.
2. Insights from this meta‐analysis will inform emerging development, adaptation, and pilot‐testing of IPV digital interventions that augment traditional modalities provided by interdisciplinary experts with violence prevention and digital health expertise.
3. Findings may also reinforce severe concerns for the digital priorities and needs of survivors who face technical obstacles (e.g., inequitable access to the Internet) or digital divisions along class and gender lines, as well as adverse socio‐ecological conditions that could moderate goals for intervention.
4. Technology may provide resources‐limited partner violence agencies with a cost‐effective way to provide services, making digital initiatives a vital platform to support (and investigate).

However, digital interventions are not without their challenges given concerns about their safety, accessibility, and confidentiality (Emezue, 2020). Other challenges include a noted digital divide in under‐resourced communities that hampers the access, accessibility, skillset, and willingness of survivors to use digital interventions. In addition, survivors with intellectual or cognitive impairments (e.g., older age groups and neurodiverse survivors) and people of various abilities (e.g., deaf and hard‐of‐hearing survivors) may not be readily involved with current technologies. Not to mention, offenders have devised ways to covertly track the survivor's online presence round‐the‐clock. Abusive partners pose a sophisticated obstacle to the use of digital interventions (Emezue, 2020), from using spyware to GPS monitoring to infiltrating support spaces online under false pretexts. Owing to the cost of smartphones and internet service, device inequalities often hamper access to digital support, making it impossible for low‐income and low‐tech survivors to leave abusive relationships.

## 2 OBJECTIVES

To synthesize current evidence on the intervention and treatment effects of digital and technology‐based interventions (mHealth and eHealth) addressing IPV mental health outcomes (depression, anxiety, and PTSD). This study's research questions are as follows: (a) What are the overall average treatment effects of IPV digital interventions on IPV survivors' mental health outcomes? (b) Do these mental health outcomes vary based on study methodological designs, sample characteristics, and intervention characteristics?

## 3 METHODS

### 3.1 Criteria for considering studies for this review

#### 3.1.1 Types of studies

##### 3.1.1.1 Inclusion criteria

Studies considered in this meta‐analysis include the following.

1. Randomized control trials (RCTs), where survivors are randomly assigned to control and intervention conditions.
2. Quasi‐RCTs, where a quasi‐random method of allocation is used, (e.g., the order of recruitment).
3. Observational studies (e.g., case‐control and cohort), for example, using *matching* design to establish group equivalence.
4. Quasi‐experimental studies (or controlled clinical trial, no randomizations, e.g., interrupted time series).

a. With participant matching to control for confounders.
b. With repeated measures or controlled before‐after studies.

Notably, ethical and safety concerns may preclude the randomization of IPV survivors to specific intervention conditions in the face of life‐or‐death IPV situations. Therefore, we will not exclude studies based on a lack of random assignment. All studies will measure IPV‐related mental health as an outcome and provide outcome data to calculate effect sizes for PTSD, anxiety, depression, and victimization (physical, psychological, and sexual violence). Studies will be full‐text accessible, published in any language (translatable into English), in published or unpublished repositories, published since January 2009 (to account for the advent of the proliferation of mobile phones and mHealth interventions in the last decade; see Anderson‐Lewis et al., 2018). Studies in which IPV outcomes are secondary will be included, provided the authors explicitly state an intention to change IPV victimization outcomes. Studies may use technology‐based modalities to deploy their intervention (e.g., mHealth, eHealth, and telehealth). For clarity, eHealth encompasses the safe and efficient use of ICT to support healthcare professionals and patients in health‐related fields. In contrast, mobile health (mHealth) is a branch of eHealth involving mobile devices to deliver or use health care, exchange clinical information, and collect data. Telehealth requires the use of ICT for healthcare practitioners to provide healthcare services; it facilitates a secure sharing of information, allowing patients to communicate health‐related concerns such as prevention, diagnosis, treatment, and remote follow‐up while addressing logistics and distance concerns.

Interventions may occur in any setting, including in a clinical or medical setting, school, mental health, emergency department, home‐visitation, or other community‐based settings. No limitations will be placed on the country of study, for example, low and middle‐income countries or regionality, such as rural versus urban localities. Studies may include any comparator/control groups in the form of usual care, waitlist controls, placebo, or alternative control formats.

##### 3.1.1.2 Exclusion criteria

Common reasons for study exclusion will be non‐IPV studies (e.g., child abuse, peer violence, acquaintance rape, elder abuse, bullying, parental violence, and other nondating victimization). Studies will also be excluded if the extent of technology use in the study is limited to participant randomization, recruitment (e.g., automated study registration websites), screening only, follow‐up only, and were in effect not targeting any IPV mental health outcomes using technology. Studies will also be excluded if they are without a clear characterization of IPV outcomes. Pilot studies that focused on feasibility and acceptability are excluded.

#### 3.1.2 Types of participants

The population of interest will be participants of all genders and ages who are experiencing or have experienced IPV. Most IPV interventions emphasize secondary and tertiary prevention (i.e., intervention after violence has occurred) and focus on reproductive‐age women (14–46 years). Given this locus, we expect studies with mostly adults (>18) female‐identifying survivors. Dating violence occurs among adolescents (at least as young as 13 years). Therefore, attempts will be made to capture studies on adolescent dating violence interventions provided the violence occurs in the context of partner violence (e.g., dating violence). Also, since IPV victimization is gender nonspecific, studies with survivors of all genders experiencing partner violence will be considered. Studies may consist of survivors who are racial/ethnic minorities, gender/sexual minorities, pregnant or infertile, justice‐involved women, rural, single young mothers, HIV‐positive, and survivors from low‐ or middle‐income countries. All such participants are of interest. Studies lacking pertinent disaggregated data will be excluded, and authors may be reached for clarifying data. Eligibility decisions will keep faith with the objectives of this review, in addition we have a wide catchment area with exclusion limited to type of violence. No exclusion age, gender, ethnicity, setting, and so forth. Sensitivity analyses may help assess the impact of these characteristics on effect sizes, if any.

#### 3.1.3 Types of interventions

##### 3.1.3.1 Experimental intervention

We will consider all types of IPV digital interventions (mHealth, eHealth, and telehealth) designed to reduce IPV and related mental health outcomes. Operationally, IPV digital interventions are those deployed via mHealth, eHealth, telehealth, and personalized digital platforms. We will consider interventions incorporating IPV‐related mental health outcomes as a primary or secondary outcome. Interventions with digital‐only or digital plus traditional modality will be included. Digital interventions may be used for added functions, such as safety planning, digital consultation, referral‐to‐care, psychoeducation, prevention of IPV and related morbidities, but must ultimately target mental health outcomes that are measured in each study. For example, the study by Koziol‐McLain et al. (2018) used a three‐component decision aid intervention, involving (a) safety priority setting activities in a multicriteria decision model; (b) accessed risk awareness using the DA (Campbell, 2002; Campbell & Glass, 2009) or DA‐Revised (for females in same‐sex relationships); and (c) created an individual tailored safety plan (Koziol‐McLain et al., 2018).

##### 3.1.3.2 Control or comparator intervention

Control conditions can involve UC, intervention‐as‐usual, waitlist controls, an active placebo control group. An example of a control measure is a smartphone app or website containing standard, nonindividualized information on IPV (see Koziol‐McLain et al., 2018), or a psycho‐educational self‐help website with a standardized list of IPV resources or a standardized emergency safety plan (Littleton et al., 2016). In some studies, the control group may receive the same modular psychoeducation as the intervention group (i.e., education then referral to care) but not in a digital format. In others, the control group may receive traditional modalities such as face‐to‐face counseling (i.e., enhanced usual care) by a healthcare provider (Constantino et al., 2015).

#### 3.1.4 Types of outcome measures

Two classes of outcomes are of interest in this meta‐analysis as common targets of IPV digital interventions: (a) mental health outcomes (see Primary outcomes) and (b) psychosocial outcomes (see Secondary outcomes). Outcomes may be measured using self‐ or partner‐reports, as well as with validated instruments. Common examples of validated instruments may include, *Revised Conflicts Tactics Scale* (CTS‐2; see Straus, Hamby, Boney‐McCoy, & Sugarman, 1996), the *Center for Epidemiologic Studies‐Depression* (CES‐D; see Radloff, 1977, or CES‐D revised, see Eaton, Smith, Ybarra, Muntaner, & Tien, 2004) and the *PTSD Checklist* (Blanchard, Jones‐Alexander, Buckley, & Forneris, 1996); and the *DA* tool (Campbell, 2002; Campbell & Glass, 2009); all with strong convergent validity to other similar measures. We will report psychometric properties (validity and reliability) of outcome measurement instruments. The study's choice of outcome measures will not influence inclusion in this meta‐analysis, as outcome measures are study‐defined and so challenging to hypothesize prior to coding.

##### 3.1.4.1 Primary outcomes

The primary outcomes of interest to be quantitatively synthesized are as follows.

1. IPV‐related mental health outcomes commonly targeted via interventions:

a. Depression, or any clinical/base change in depressive symptoms.
b. PTSD, or any clinical/base change in PTSD symptoms.
c. Anxiety, or any clinical/base change in anxiety symptoms.

2. Any changes in *IPV victimization* (and perpetration) outcomes including *frequency* and *severity* of IPV experiences (not limited to physical aggression, sexual violence, psychological abuse, coercive behaviors, and verbal abuse). Both frequency and severity of IPV victimization contribute to PTSD, depression, and anxiety (Dutton et al., 2006).

##### 3.1.4.2 Secondary outcomes

The secondary outcomes of interest may include the following.

1. Increase in self‐efficacy (or ability to create and use a safety plan based on intra‐person, demographics, and extra‐person variables). A general sense of personal competence (i.e., general self‐efficacy) and relationship self‐efficacy (RSE) are expected outcomes typically measured using self‐rated or validated instruments such as The RSE Questionnaire (Lopez & Lent, 1991).
2. Increase in risk awareness (or objective awareness of present or future IPV risk), measured using actuarial surveys.
3. Reduction in decisional conflict (ability to navigate an abusive context, stay or leave the abusive relationship).
4. Increase in safety planning (e.g., executing a personalized safety plan).

In sum, secondary outcomes may be psychosocial, socioeconomic, or somatic. In IPV intervention studies, use a combination of self‐reports and actuarial surveys, making it problematic to quantitatively pool these outcomes together owing to the wide variety in how they are measured from an a priori review of the literature. Where further clarification of study outcomes is warranted, study authors will be contacted no more than twice to provide clarification. The primary and secondary outcomes will be narratively described in the full review, with additional quantitative meta‐analysis performed on all primary outcomes. Other potential outcomes will be added early in the coding process should this be warranted by the data collected.

##### 3.1.4.3 Duration of follow‐up

No limitations will be placed on studies by their duration of follow‐up (or follow‐up as part of the intervention dosage). However, where the duration of follow‐up is highly divergent across studies enough to influence intervention effects significantly, we will categorize studies into three subgroups for analysis: short‐term (baseline to 3 months), medium‐term (3‐6 months), and long‐term (>6 months). This will provide evidence on the temporal effects of the IPV digital interventions on survivor mental health outcomes.

### 3.2 Search methods for identification of studies

An initial retrieval set of articles will be used to create a library of Medical Subject Headings (MeSH) terms using an online search building tool called Yale MeSH Analyzer (see Grossetta & Wang, 2018). The MeSH term analysis grid will be generated to help establish indexing consistency. Going forward, key search terms will be combined with MeSH or Emtree headings (unique to EMBASE) for two major classifications: “digital interventions” AND “intimate partner violence.” For the purpose of this review, we include relevant forms of *domestic violence* and *IPV* in our search strategy as both terms are often conflated in the literature. All search terms will be restricted or expanded using suitable Boolean and proximity operators using sensitivity‐ and precision‐maximizing modalities. Filters and limiters (where applicable) may include “English” and “year of publication.” Controlled vocabulary will be used to account for variant spellings, truncations, and wildcards. Database‐specific searches will be done using aggregated or simplified keyword (free‐text and subject headings):

An example of an aggregated search using the boolean configuration for “technology” AND “intimate partner violence”:

**Table:** 

In traditional and grey databases			
	Technology		Intimate partner violence
*Search terms*	(mHealth OR mobile apps OR eHealth OR mobile applications OR interactive mobile application OR apps OR "mobile app" OR electronic interventions OR safety app OR decision aid OR mobile intervention OR smartphone OR smartphone apps OR smartphone‐based app OR smartphone‐delivered OR mobile‐delivered OR technology‐mediated OR internet‐based OR web‐based OR computer‐based OR computerized OR electronic OR technology‐based OR "use of technology" OR m‐Health OR e‐Health OR social network OR social media OR mobile phone OR mobile device OR Virtual communit* OR virtual reality OR Twitter OR Facebook OR WhatsApp OR WeChat “mobile health” OR “mobile care” OR “m Health” OR “mobile phone” OR “mobile device” OR “mobile technology” OR “mobile communication” OR “mobile telecommunication” OR “mobile app” OR “mobile application” OR “mobile tool” OR “mobile messaging” OR “mobile electronic device” OR “mobile telephone” OR “mobile phones” OR “mobile devices” OR “mobile technologies” OR “mobile communications” OR “mobile telecommunications” OR “mobile apps” OR “mobile applications” OR “mobile tools” OR “mobile messages” OR “mobile electronic devices” OR “mobile telephones” OR “mobile intervention” OR “mobile interventions” OR “mobile delivered” OR “mobile delivery OR information, communication technology OR ICT OR email)	AND	("Intimate Partner Violence"[Mesh] OR "partner violence" OR "partner abuse" OR "dating violence" OR "dating abuse" OR dating violence OR partner abuse OR adolescent dating violence OR OR stalking OR assault OR coercion OR "digital abuse" OR rape OR battered women OR "domestic abuse” OR “wife abuse" OR "Spouse Abuse”[Mesh] OR “Domestic Violence”[Mesh:noexp] OR intimate partner violence[tiab] OR domestic violence[tiab] OR dating violence[tiab] OR partner violence[tiab] OR domestic abuse[tiab] OR partner abuse[tiab] OR (Abuse[tiab] OR abusive[tiab] OR abused[tiab] OR battered[tiab] OR battering[tiab] OR violent[tiab] OR violence[tiab] OR assaultive[tiab])

#### 3.2.1 Electronic searches

A comprehensive search will be conducted in several databases for national and international studies since 2009 (coinciding with the proliferation of mobile devices and mHealth) with the support of a health science research librarian.

1. Traditional broad‐spectrum databases:

a. PubMed Central (PMC).
b. Cochrane Central Register of Controlled Trials (CENTRAL).

i. MEDLINE/PubMed (using the Cochrane Highly Sensitive Search Strategy for identifying randomized trials in MEDLINE),
ii. EMBASE.

c. Web of Science.
d. Scopus.
e. Cumulative Index of Nursing and Allied Health Literature (CINAHL) Plus.
f. PsychINFO.

2. National and international clinical trial registries (e.g., ClinicalTrials.gov, NIH rePORT, and the Australian New Zealand Clinical Trials Registry, and World Health Organization International Clinical Trials Registry Platform).
3. "Gray literature" databases: Google Scholar, ProQuest Dissertations and Theses Global, preprint databases (e.g., PsyArXiv and medRxiv).
4. Open‐source specialty journals such as the Journal of Medical Internet Research, Digital Health, and Frontiers in Digital Health. We will search the ACM Digital Library (covering computing and information technology), Health Technology Assessment Database, Compendex, Global Index Medicus, and The Institute of Electrical and Electronics Engineers databases (e.g., IEEE Xplore Digital Library), known for collating digital interventions for social and humanitarian causes.
5. Gender violence observatories:

a. Women's Studies International Forum.
b. Family and Society Studies Worldwide.
c. VAWnet (National Resource Center on Domestic Violence).

#### 3.2.2 Searching other resources

1. Topic‐related organizational websites (e.g., www.VAWnet.org) will be searched to reduce possible publication bias, as well as purposive searches of key authors predominantly involved in mHealth‐linked IPV interventions and their concurrent publications on this topic.
2. In addition, searches will be conducted by hand‐searching recent reviews of IPV interventions for digital interventions.
3. Reference searching of all included studies for further relevant studies.
4. Lastly, prominent author publications on mHealth‐linked IPV interventions will be searched by name for studies that meet criteria.

### 3.3 Data collection and analysis

#### 3.3.1 Description of methods used in primary research

We anticipate IPV digital interventions will use randomized control trials (parallel, longitudinal, and clusters), quasi‐experimental studies (e.g., single group pre‐ and posttest designs). Various forms of randomization will be considered for inclusion. We anticipate study data analysis plans to control for potential confounding variables and compliance and attrition issues (e.g., using intent‐to‐treat protocols analyzing each participant regardless of compliance). Experimental studies may compare baseline characteristics of intervention completers to dropouts. Studies may include IPV as a principal outcome or secondary outcome in addition to treatment for other conditions, such as HIV/STI, substance abuse, trauma distress (e.g., state and trait anger), mental health burden, as well as somatic morbidities. However, of note, where IPV intersects with other comorbidities such as alcohol and substance use, Screening, Brief Intervention, and Referral to Treatment (SBIRT) model have been used for mental health and substance abuse (Gilbert et al., 2016). Due to IPV's subjective and personal nature, meaning‐forming and exploration of realistic lived experiences have warranted qualitative methods. For example, in the nested qualitative interpretive study among women who use drugs in Kyrgyzstan (Bacchus et al., 2016). Adult and adolescent female survivor interviews and focus groups are typical where the aim is to understand the feasibility and acceptability of IPV digital interventions (Alhusen et al., 2015; Bloom et al., 2016; Debnam & Kumodzi, 2019; Lindsay et al., 2013).

#### 3.3.2 Criteria for determination of independent findings

1. Multiple studies on a single group of participants will be treated as one study to minimize duplicated data bias and overestimation of intervention effect with the exception of any important distinctions (e.g., new data not previously published).
2. A primary publication with different studies will be extracted separately provided samples are independent and not nested.
3. We will consider multiple time points of outcomes measures (3, 6, and 12 months) separately for this meta‐analyses.
4. If multiple publications for the same data are sourced, only the most comprehensive report of the data will be used.
5. Where needed, we will combine groups to create a single pair‐wise comparison.
6. Effect sizes from studies that report similar outcomes will be averaged using the method described by Borenstein, Hedges, Higgins, and Rothstein (2009).
7. No determination of study independence will not be based on authoring activities. (i.e., for example, the same author(s) across studies) as long as other criteria are met.

#### 3.3.3 Selection of studies

At least two reviewers (including a leading dating violence researcher) along with a health science research librarian will independently screen for titles and abstracts from a random selection of yielded articles. For consistency, an interrater agreement of 80% (range of 0–1) using Kappa statistics for ordinal data (i.e., relative observed agreement, Landis & Koch, 1977) and 80% intraclass correlation coefficient (ICC) statistic (range of 0–1) for continuous variables will be established for (a) study selection; (b) data/outcome extraction; and (c) risk of bias assessments. Ongoing study selection will be continued, and studies outside our criteria will be excluded (e.g., duplicates, non‐IPV studies). Reasons for study exclusion will be presented in the full review.

#### 3.3.4 Data extraction and management

At least two reviewers will independently extract data from studies to mitigate data extraction errors. Having reached interrater consistency, one reviewer will recover the full‐texts of relevant studies. Both reviewers will independently evaluate full‐text (and other data sources) based on set guidelines. A data extraction form and coding sheet (hierarchical and systematic coding structure) will be created to include all relevant codable variables as found in yielded studies as well as prevent low data entry and transcription errors. The coding sheet will be pilot‐tested with a random subset of studies. Then a codebook will be created to guide this process (Brown, Upchurch, & Acton, 2003).

Extracted data will include:

1. Study details (author(s), title, year, type of research design, journal of publication, trial status, sample size, study setting).
2. Study characteristics (sample size, study design, number of events and participants per group, mean score, and standard deviations).
3. Participant characteristics (age, gender, socioeconomic status, race/ethnicity, sexual orientation, and other relevant characteristics).
4. Intervention descriptors (e.g., assignment and blinding protocol, type of mHealth/eHealth intervention, dose, time period).
5. Control or comparison descriptors (e.g., usual care or nondigital platforms).
6. Inclusion and exclusion criteria (e.g., the sampling frame for exclusion from the intervention).
7. Primary and secondary outcomes data (e.g., effect sizes, outcome type, outcome measurement instrument).
8. Study fidelity outcomes (attrition, intervention completion, follow‐up).
9. Study results (prevalence n/N (%); proportion, 95% CI), limitations, and implications.

For internal validity, all studies will be double‐coded by an independent member of the review team. Data extraction discrepancies will be discussed until consensus. Extracted data will be reported in the *Characteristics of included studies* table.

#### 3.3.5 Assessment of risk of bias in included studies

This meta‐analysis will report on the strength of study evidence by analyzing the risk of bias using the Cochrane risk‐of‐bias tool for randomized trials, Version II (or RoB 2). RoB 2 uses a fixed set of domains of bias, focusing on aspects of trial design, conduct, and reporting. This meta‐analysis will also use Cochrane's ROBINS‐I (Risk of Bias in Non‐Randomized Studies‐of Interventions) to assess the risk of bias in nonrandomized studies (Sterne et al., 2016). The risk of bias analysis will prevent bias due to underestimated or overestimated study‐level effects (Sterne et al., 2019; Higgins et al., 2011). Both tools (RoB 2 and ROBINS‐I) use a series of “signaling questions” to elicit information about study risk of bias across a finite list of domains. An algorithm‐generated judgment about the risk of bias is provided based on answers to the signaling questions (Sterne et al., 2019). This process will be conducted by at least two reviewers who will discuss and resolve discrepancies.

##### 3.3.5.1 Cochrane risk‐of‐bias tool for randomized trials, Version II (or RoB 2)

For RoB 2, the risk of bias will be reported based on the following fixed classification scheme:

1. Domain 1: Risk of bias arising from the randomization process.
2. Domain 2a: Risk of bias due to deviations from the intended interventions (effect of assignment to intervention) if “intent‐to‐treat.”
3. Domain 2b: Risk of bias due to deviations from the intended interventions (effect of adhering to intervention) if “per‐protocol” analysis.
4. Domain 3: Risk of bias due to missing outcome data.
5. Domain 4: Risk of bias in the measurement of the outcome.
6. Domain 5: Risk of bias in the selection of the reported result.

We rate overall risk‐of‐bias judgment as follows:

1. A “low risk of bias” if the study is judged to be at *low risk* of bias for all domains.
2. An “unclear risk” study is judged to *raise some concerns* in at least one domain, but not to be at *high risk* of bias for any domain.
3. A “high risk” study is judged to be at *high risk* of bias in at least one domain in a way that substantially lowers confidence in the result.

##### 3.3.5.2 Cochrane's ROBINS‐I

1. Specify the research question through consideration of a target trial.
2. Specify the outcome and result being assessed.
3. For the specified result, examine how the confounders and cointerventions were addressed.
4. Answer signaling questions for the seven bias domains.
5. Formulate risk of bias judgments for each of the seven bias domains, informed by answers to the signaling questions.
6. Formulate an overall judgment on the risk of bias for the outcome and result being assessed.

We rate overall risk‐of‐bias judgment as follows:

1. Judgments for each bias domain will indicate “Low,” “Moderate,” “Serious,” or “Critical” risk of bias. In addition, the risk of bias will be assessed in view of confounders (e.g., differences in exposure to partner violence) and cointerventions (e.g., information therapy from filling out baseline surveys or viewing brochures at study sites).

A sample of 10 studies spanning different levels of risk of bias (low/high/some concerns) will be pilot‐coded and compared among authors to test fidelity with a bias classification scheme (Higgins, Deeks, & Altman, 2008; Sterne et al., 2019). Discrepancies will be resolved via discussion. As needed, an experienced health science statistician will be available for ongoing consultations. Each study's risk of bias assessments will not be used to decide inclusion in the meta‐analysis. Of note, IPV interventions are time‐ and safety‐sensitive. Thus, methodological heterogeneity may vary, given that participant familiarity with digital intervention may impact intervention effects considering the novelty and self‐management approach of digital interventions (Higgins et al., 2011). Given this, it is not unlikely a good number of studies may face exclusion, preventing the robustness of the evidence. However, we will explore the stratification of the meta‐analyses based on low, unclear, or high risk of bias as recommended by Higgins et al. (2011) (see Data synthesis plan). The overall risk of bias analysis will be reported in the “Risk of bias” table.

#### 3.3.6 Measures of treatment effect

An effect size will be calculated for each primary outcome (see Types of outcome measures). Relevant summary data will be extracted to calculate effect sizes using the RevMan software program and calculator as follows.

##### 3.3.6.1 Continuous data

Effect estimate for continuous outcomes will be quantified as the standardized mean difference (SMD). Proportions will be converted using logit transformation to be reported as a percentage with its 95% CI. The SMD is expressed in standard deviation units to account for adjusted and unadjusted means as well as divergent outcomes measures. Further, SMD is a suitable statistic than the mean difference (MD) when outcome measures vary across studies (Higgins et al., 2016). Relevant effect size indices to calculate average effect sizes will be extracted from trials (i.e., means, sample sizes, SD). Other natural (or raw) units will be transformed to SMD using change‐from‐baseline scores. Hedges' *g* will be the primary index of choice to compute the SMD between groups, considering its suitability for small sample sizes less than 20 (Hedges & Olkin, 1985; Hedges, Tipton, & Johnson, 2010). The inverse‐variance DerSimonian‐Laird random‐effects model for continuous data is used as a computational model as programmed into RevMan. Where needed, Hedges' *g* will be computed from test statistics, such as Cohen's *d*, the *t*‐ and F‐statistic, *χ* ^2^ values, correlation coefficients with their corresponding *p* values (Borenstein et al., 2009). Effect sizes based on correlational data will be analyzed in Fisher's *z* units (Borenstein, Hedges, Higgins, & Rothstein, 2011). In order to show the range of effects in future research, the 95% prediction intervals will be calculated.

##### 3.3.6.2 Dichotomous and discrete data

For dichotomous outcomes (*yes* or *no, true* or *false* answers to IPV experience), we will use the relative risk ratio (RR) measure of effect size having extracted relevant data (Higgins et al., 2008). RR (along with 95% CIs) will be used to compare the risk of IPV frequency in the treatment groups against the risk of IPV in the control group. Unlike the RR, the odds ratio (OR) may not apply if the intervention is reducing IPV risk and not fully eradicating IPV risk (i.e., *events vs. nonevents*). Therefore, appropriate transformations will convert effect sizes to a standard metric (e.g., OR to RR) pending findings from data extraction. The random‐effects model used for dichotomous data will be the Mantel‐Haenszel random‐effects method (Mantel & Haenszel, 1959, Greenland & Robins, 1985)—particularly if data is sparse—as programmed into Revman (see Cochrane Handbook; Higgins et al., 2011). From a practical point of view, IPV is unlikely to be measured as a dichotomous outcome, since doing this will limit the scope of prevalence data captured using binary methods. In addition, while definitions of IPV may vary (Costa & Barros, 2016), this conceptual diversity is ignored as we expect useful convergent validity across IPV instruments from a practical point of view (e.g., for example, a measure of physical violence should capture positive responses to hitting, slapping, punching, strangulation). Outcome measures using ordinal scales (e.g., Likert scales) will be meta‐analyzed as dichotomous data. As needed, we will re‐express ORs as SMDs (and vice versa).

##### 3.3.6.3 Dependent effect sizes

Dependent effect sizes occur for multifactorial reasons (Van den Noortgate, López‐López, Marín‐Martínez, & Sánchez‐Meca, 2013). To avoid biasing the average effect size, we will pool the intervention group means and standard deviation for multiple treatment conditions (e.g., [smartphone arm vs text message arm] vs. [control arm]) prior to calculating the effect size for studies that include multiple intervention arms with only one control group. This review will note instances of effect size dependence, such as multiple outcomes measured by the same set of participants, outcome measured at multiple follow‐up times, and multiple correlations from a common sample. These issues will be handled using known formulas by Gleser and Olkin (2009) and as recommended by the Cochrane Handbook (Higgins et al., 2011). Due to the inclusion of multiple study types, we apply the Borenstein et al. (2009) method to calculate a weighted overall mean effect of different types of interventions compared with a matched control group where sample sizes, means, and standard deviations are known. For example, we will combine multiarm trail groups to create a single pair‐wise comparison (see Cochrane Handbook, ch. 16; Higgins et al., 2011). When multiple effect size estimates are generated from the same outcome, effect sizes will be averaged. An effect size of 0.2 will be considered a small effect, 0.5 a moderate effect, and 0.8 a large effect as recommended by Cohen (1988).

#### 3.3.7 Unit of analysis issues

We expect individually randomized trials with participants randomly allotted to intervention or control groups. In the absence of individual randomization (e.g., cluster‐randomized trials, cross‐over trials, and repeated measures) we will apply statistical adjustments to correct for unit‐of‐analysis issues according to the Cochrane manual (Higgins et al., 2011). For example, the adjustment may include log transformations (the use of log RRs and standard errors (SEs) of log RRs) for both individual and cluster‐randomized trials to prevent the overestimation of the intervention effects (e.g., narrow confidence intervals or small SEs; see Pigott, 2012; Thomas, Ramsay, McAuley, & Grimshaw, 2003). We will estimate the ICC for cluster‐randomized trials to reduce trials to their effective sample size (i.e., a standard metric) for RevMan analysis. Where we find cross‐over trials, data from the first treatment period will be used. We find multiple comparator groups, trials will be analyzed separately by the comparator group. Where the duration of follow‐up is a stratifying factor, these will be separately analyzed as defined: short‐term (0–3 months), medium‐term (3–6), and long‐term follow‐up (>6 months). We will explore the impacts of these issues using Sensitivity analysis.

#### 3.3.8 Dealing with missing data

Where data is unclear or missing to calculate the effect sizes, study authors will be contacted for further clarification and to obtain relevant data using clarifying questions as recommended by the Cochrane Handbook for Systematic Reviews of Interventions (Higgins et al., 2011). Authors of studies that presented data in a graphical format will be contacted to obtain exact values. Studies with data omitted will be included in this review and meta‐analysis with considerations for any bias introduced as a result of this inclusion studies via Sensitivity analysis to explore the effects of these missing data on intervention effects. In the absence of sufficient data, we will not exclude studies. Strategies to address missing data according to the Cochrane Handbook may include the use of conservative data imputation, such as *d* = 0, and *p* = .05, or a literature‐derived population standard deviation to account for imputation with uncertainty (Higgins et al., 2011). Another strategy will be to use statistical models to allow for missing data making assumptions about their relationships with the available data (see Cochrane Handbook for Systematic Reviews of Interventions; Higgins et al., 2011).

#### 3.3.9 Assessment of heterogeneity

##### 3.3.9.1 Methodological and clinical heterogeneity

Clinical heterogeneity and methodological heterogeneity will be subjectively considered and reported in a narrative review format. We will first qualitatively assess and report methodological (heterogeneity in study design) and clinical heterogeneity (heterogeneity of survivors, interventions, and outcomes). For example, we will consider if digital interventions are different in important ways across studies based on study‐specific comparisons. Further, it is likely digital interventions (eHealth and mHealth) may differ, however, these differences do not take away from the wider question about the effectiveness of digital interventions much unlike traditional ones. Studies excluded in the meta‐analysis but useful to the systematic narrative review will be retained.

##### 3.3.9.2 Statistical heterogeneity

Statistical heterogeneity (heterogeneity in the effects across study due to random error) is inevitable in behavioral health meta‐analyses attributable to differences in survivors, intervention types, control measures, duration of follow‐up, study outcomes, and settings (Higgins et al., 2011). Therefore, a test for statistical heterogeneity will be conducted alongside a narrative review. A random‐effects meta‐analysis will be utilized since (a) we assume an approximately normal distribution; (b) we assume our search may not capture all available studies (Lipsey & Wilson, 2001); and (c) we anticipate varying true effects across studies reflective of the “real‐world” ecology of IPV survivors (DerSimonian & Laird, 1986; Lipsey & Wilson, 2001).

##### 3.3.9.3 Confirmation of statistical heterogeneity (Cochran's *Q*)

We will report the standard *χ* ^2^ test (Cochran's *Q*‐statistic) with the accompanying *I*² to test the null hypothesis that effects across studies are the same (Hedges & Olkin, 1985). Conceptually, Cochran's *Q*‐statistic reflects divergences between each study's effect against the overall mean effect. Cochran's *Q* test for heterogeneity is well‐suited for fixed‐effects models but is applicable to random‐effects analyses (used in this meta‐analysis). Within the random‐effects model, Cochran's *Q*‐statistic tests the statistical significance of τ^2^, the variance component for the model. We will report the *p* value for this *χ* ^2^ test using a recommended *p* < .01 significance level due to this test's low power (Sutton, Abrams, & Jones, 2000). If the number of studies included is less than 20, Cochran's *Q*‐statistic will be interpreted with a caveat.

##### 3.3.9.4 **Extent of heterogeneity (Higgins** *I* **² and Kendall's τ²)**

The degree of heterogeneity will be calculated using the *I*² statistic (ranges from 0% to 100%), which is a percentage of variation across studies (Deeks, Higgins, & Altman, 2008). *I*² does not depend on the number of studies. We anticipate high *I*² due to inherent heterogeneities. *An I*² threshold of 50% will be used to identify moderate heterogeneity according to recommendations by Higgins & Thompson, 2002. The pooling of outcomes across studies/subgroups would be appropriate only if heterogeneity is *moderate* as specified. In addition, between‐study variance will be reported using Kendall's τ² (or tau‐squared) estimated using maximum likelihood procedures (Cheung, 2013). That is, the higher the τ², the higher the variance (Borenstein et al., 2011; Deeks et al., 2008). τ² is an absolute value and will be considered low at (0.33), moderate at (1.0), or high at (3.0; Borenstein et al., 2009).

##### 3.3.9.5 Strength of heterogeneity

The noncentral *χ* ^2^ distribution test of homogeneity to determine the strength of heterogeneity will be reported to test the strength of between‐study variance (Higgins et al., 2008; Lau, Ioannidis, & Schmid, 1997).

##### 3.3.9.6 Visual evaluation of heterogeneity

Preliminary analysis using forest plots will note the width and overlaps between confidence intervals around the study effect parameter. Both forest plots and L'Abbé plot will be used to visualize the extent of heterogeneity and inconsistencies across studies as well as the significance of the pooled effect (Song, 1999; L'Abbe, Detsky, & O'Rourke, 1987). Unclear data and outlier outcomes will be reported narratively. The sources and extent of heterogeneity will be handled as follows: (a) using a random‐effects model; (b) by reporting a summary estimate of a location parameter (e.g., summary standardized mean difference estimate) and a variability parameter (i.e., summary 95% CI); (c) possibly reporting a 95% prediction interval if most studies report a low risk of bias (Higgins et al., 2011); and (d) we will consider potential moderators (e.g., survivor's age) to understand the impact on heterogeneity. Reasons for heterogeneity will be further explored using subgroup analysis.

#### 3.3.10 Assessment of reporting biases

Funnel plots will illustrate each effect size (horizontal axis) plotted against its standard error (*y*‐axis in a reversed scale) or precision (1/standard error; Lau et al., 1997). An asymmetrical funnel plot will have a statistically significant test confirming reporting biases. Whereas a symmetrical funnel plot indicates a low risk of publication bias. To further test the asymmetry of the funnel plot, an Egger's asymmetry test of the intercept will be reported with a *p* > .05 significance level (Egger, Smith George, Schneider, & Minder, 1997; Sterne & Egger, 2005). If publication bias is suspected, then a “trim and fill” method will adjust for symmetry by the iterative addition and removal of studies to gauge the impact on the overall effect. Pending a minimum number of studies (*k* ≥ 10) a possible metaregression will be conducted as described in Section 3.3.13.

#### 3.3.11 Data synthesis

To summarize the overall effect estimate of the IPV digital interventions, meta‐analyses will be analyzed using separate comparisons using the RevMan manager. The software will undertake random‐effects meta‐analyses along with assessments of heterogeneity using DerSimonian and Laird's methodology as programmed in RevMan (DerSimonian & Laird, 1986). A random‐effects model will account for differences in true effect across studies, this will depend on the number of studies included (*k*), within‐study sample sizes, and ultimately provide a parsimonious estimate of the overall effect size estimate (Borenstein et al., 2011; Valentine, Pigott, & Rothstein, 2010). Heterogeneity is assessed using *I* ^2^ and *χ* ^2^ measures of heterogeneity (Cochran's *Q* test; 95% CI), as well as graphical plots (forest plots and L'Abbé plot). Reasons for heterogeneity will also be explored using subgroup analysis. The pooling of outcomes across studies would be appropriate only if heterogeneity is *moderate* (≤50%).

Where data permits, effect sizes will be calculated separately (pending number of studies for each outcome) using a series of pairwise comparisons, for example, by nonrandom assignment, age (young vs. old), risk of bias, duration of follow‐up, and digital intervention features (mHealth vs. eHealth) as follows.

- By *outcome types*, we will report a separate weighed analysis of the effects of IPV digital intervention on depression, anxiety, and PTSD outcomes.
- By random and nonrandom assignment. We will pool natural logarithms (log RRs for individually randomized trials and adjusted log RRs) for cluster‐randomized trials, and report a pooled SMD estimate for randomized trials. For pre‐ and posttest studies, we compare pretest measures matched to immediate posttest (with the possibility of including follow‐up effect sizes, if practical). The exception will be where we stratify analysis by duration of follow‐up.
- By *risk of bias*, we will stratify the meta‐analyses based on low, unclear, or high risk of bias to obtain a summary estimate of the treatment effect based on study quality as recommended by Higgins et al. (2011).
- By digital intervention features (mHealth vs. eHealth), if possible.

For each pool of summary estimates, we will report on heterogeneity, as well as a test for an overall effect. These will be on the basis of a random‐effects meta‐analysis. All pooled effects will be interpreted with the parsimonious reporting of both the 95% summary estimates and the 95% prediction intervals. Summary tables will be used where appropriate with narrative descriptions of findings. We plan to use a Type I error rate set at a significance level of .05. Where appropriate, subgroup will be compared using the analysis of variance‐like analysis to estimate the difference in intervention effect between subgroups.

If appropriate, a univariate random‐effects metaregression (where *k* ≥ 5, and each study contributes ≤1 ES estimate) will be performed to observe the impact of continuous and dichotomous explanatory variables on intervention effects where heterogeneity is in excess (≥50). Potential moderators for consideration include study design (RCT vs. non‐RCT), study duration (short through long‐term follow‐up), participant characteristics (e.g., age, sample size), quality of the studies, and intervention features (mHealth vs. eHealth). A multivariate metaregression will be conducted for study outcomes and intervention features, where possible, using a random or mixed‐effects model. Metaregression will be observational in nature without any causal inferences but will help clarify the impact of study‐level covariates on the summary effect estimates, as well as the impact of certain decision‐making parameters, such as mixed‐age interventions (i.e., older and younger participants using new technologies). Where possible, findings will be stratified into meaningful narrative categories.

#### 3.3.12 Subgroup analysis and investigation of heterogeneity

Hypothesis‐generating subgroup analyses will be performed if heterogeneity is substantial and there are a minimum of two studies per subgroup. We anticipate study heterogeneity (in terms of intervention designs, participant characteristics), as well as between‐ and within‐study variances. Subgroups analyses will explore potentially explanatory factors that are likely to vary meaningfully across studies, hypothetically.

1. Pair‐wise comparisons: randomized studies (intervention‐control) and nonrandomized studies (pretest vs. posttest).
2. Study risk of bias (low, unclear, and high risk of bias), due to anticipated variability in study methodologies.
3. Between‐ and within‐group comparison.
4. Duration of follow‐up; short (0–3 months), medium (3–6) and long‐term follow‐up (>6 months) to study temporal effects of interventions.
5. Age (adult or adolescent survivors of IPV), as it is likely the impact of the intervention might be more pronounced with younger survivors than with older survivors when you factor in pre‐existing technological/digital literacy.

A comprehensive plan for the investigation of heterogeneity is reported in Section 3.3.9.

#### 3.3.13 Sensitivity analysis

We will conduct a sensitivity analysis of study peculiarities during the search and coding process to ascertain the robustness of the results. We may explore the impact of procedural decisions (e.g., using different meta‐analysis models, fixed‐ vs. random‐effects models) and report stratified meta‐analyses based on the risk of bias (low, unclear, or high risk) as recommended by Higgins et al. (2011). We will further explore the impact of removing high‐risk (outlier) studies judged to be at *high risk* of bias in at least one domain; See the *Cochrane Handbook for Systematic Reviews of Interventions (*Higgins et al., 2011). We will compare subgroup analysis, where relevant, using the *Q* test for heterogeneity to report the difference in mean effect between compared subgroups, using a separate estimate of τ² for each subgroup if we find true between‐studies dispersion. We will explore the impact of procedural and study‐level factors in a meta‐regression (see notes under Data synthesis) to understand the impact of randomization and the input of study‐level covariates to the average effect estimate (Lau et al., 1997; Thomas et al., 2003). Other factors that warrant sensitivity analyses may not be identified until after preliminary meta‐analysis. Considerable differences in meta‐analysis results across sensitivity analyses will signify a need for cautious interpretation of mean and summary effect across subgroups.

##### 3.3.13.1 Treatment of qualitative research

We will not include qualitative studies, cross‐sectional studies, and case reports as these studies are amenable to a different type of review (i.e., coping review; see Levac, Colquhoun, & O'Brien, 2010) and do not lend themselves to robust synthesized evidence of IPV mental health outcomes as a result on an intervention.

#### 3.3.14 Summary of findings and assessment of the certainty of the evidence

The Grades of Recommendation, Assessment, Development, and Evaluation Working Group (GRADE) approach to document the certainty of evidence on each main outcome will be used (Schünemann, Hill, Guyatt, Akl, & Ahmed, 2011). In RevMan the GRADEpro GDT function will support exporting yielded data to a “Summary of findings” table holding information on the following.

1. A list of all important outcomes, both desirable and undesirable.
2. A measure of the typical burden of these outcomes (e.g., illustrative risk, or illustrative mean, on control intervention).
3. The absolute and relative magnitude of effect (if both are appropriate).
4. Numbers of participants and studies addressing these outcomes.
5. A grade of the overall quality of the body of evidence for each outcome (which may vary by outcome).
6. Space for comments (see the GRADE handbook, and *Cochrane Handbook for Systematic Reviews of Interventions*, Higgins et al. 2011; Schünemann et al., 2011).

## CONFLICT OF INTERESTS

Chuka N. Emezue is a research assistant on a multisite study involving other authors of potentially eligible studies. Tina L. Bloom is the coauthor of a potentially eligible study.

## AUTHOR CONTRIBUTIONS

C. N. E. and T. L. B., drafted and edited the protocol; contributed to study selection, will resolve any disagreement; extracted data from trials, collecting articles, coding, synthesizing the articles; contributed to data entry and coding into review software or other forms; practical methodological aspects, study screening and coding, synthesis analysis, and report writing—with expert biostatistician at the University of Missouri; interpretation and data analysis; and draft of final review, preparation for publication, and report writing. C. N. E. developed a search strategy and search relevant databases, with the help of expert biostatistician at the University of Missouri.
